# Voluntary Hospitals and Paying Patients

**Published:** 1904-01-30

**Authors:** 


### Voluntary Hospitals and Paying Patients.

The voluntary hospital system is perhaps the greatest moral adornment of the present age. The meditative will perceive in it many virtues. Not only are the masses provided in the most efficient manner possible with the means of obtaining medical treatment in case of accident or illness, but it is also given to each individual patient when he is in a hospital to see and to feel, at a time when his mind is most open to impression, the benefits of unselfishness, of self-sacrificing labour given in his behalf freely, good-naturedly, and often without material reward. In this way the seed of compassion is carried into hearts that have long been cold, and into homes where human kindness is almost unknown. Moreover, the chance is offered to every well-disposed citizen, rich and poor alike, to take a share in the good Samaritan's work by devoting some little portion of his leisure or his money to the work of lending succour to those of his fellows who are stricken down by the way. These humanising influences are sufficiently real and well recognised to lead public opinion in this country to cherish the voluntary hospital system with a jealous watchfulness lest any harm shall come to it in the progress of time. For this reason it is interesting to contemplate the present position of the general hospitals in New York in order to derive if possible some benefit from the misfortunes which have overtaken these institutions, and to be able to avoid falling into the same pitfalls which at this moment threaten them with extinction. In New York the hospitals looked at as a whole are in a state of chaos, and it has recently become necessary for the municipal government to intervene, and to expend enormous sums of public money in order to solve the problem of caring for the sick poor on business lines. Such a catastrophe is far from being realised in this country, but none the less is it important to avoid the small beginnings that may lead to such an end. It is stated on good authority that some dozen hospitals in New York are being carried on under conditions which result in an annual deficit of £90,000. The annual reports for the past year show, among others, the following examples of excess of expenditure over income (the figures are approximate):—The Lying-in Hospital, £17,500; Mount Sinai Hospital, £8,500; the Post-Graduate Hospital, £8,500; the Presbyterian Hospital, £16,000; and St. Luke's Hospital, £5,000; a total of £55,500 for the five hospitals named. It is true that these deficiencies fall to a considerable extent under the heading of extraordinary expenditure, and therefore the actual amounts are not very striking in themselves; they merely indicate an active endeavour to bring the hospitals named up to the level of modern requirements. It may be true that, in their anxiety to have everything of the best, the authorities of certain of the institutions concerned have been guilty of some extravagance, especially with regard to bricks and mortar. But whatever the explanation is, the hospital indebtedness is not in itself sufficiently large to call for alarm. The serious aspect of the case is the circumstance that the charitable public does not appear anxious to defray the expenditure. Meanwhile the great streams of charity are flowing in other directions. This attitude of the benevolent is, no doubt, largely due to the attempt to mingle charity with business, for it is a common custom among the hospitals of New York to take in paying patients as well as those who cannot contribute anything to the expenses of their treatment. At least, this is the explanation offered by individuals who are in a position to express an opinion, and it is by no means difficult to perceive how such an influence might operate. The result of this plan is to place the hospitals concerned on such a basis that they are not looked upon by the general public as purely charitable institutions, and the loss of pecuniary support which of necessity follows is by no means compensated for by the fees received from the patients who are able to make payments. It is urged, moreover, that a tendency arises to neglect the poorest patients in favour of those who can make some contribution to the hospital funds. Whether such an accusation be true or false, and we do not for a moment believe it can be justified, it is easy to recognise the harm that must accrue from the rumour as it reaches the ears of the benevolent.

It is well to dwell on the evil results of this system, for although it does not obtain to any considerable degree in the hospitals in this country, yet its tendencies may not be sufficiently well recognised. If it be desirable to have paying departments attached to the general hospitals, they should be kept absolutely separate and distinct from the rest of the institution, so that all confusion may be avoided. There is at least one hospital in the metropolis whose burdens would be considerably lightened if the crippling policy of combining bad businefs and doubtful charity were abandoned.

